# Views among Malawian women about joining HIV prevention clinical trials when pregnant

**DOI:** 10.1186/s12981-020-00271-6

**Published:** 2020-05-27

**Authors:** Kristen Sullivan, Tiwonge Mtande, Elana Jaffe, Nora Rosenberg, Chifundo Zimba, Irving Hoffman, Maggie Little, Ruth Faden, Anne Drapkin Lyerly

**Affiliations:** 1 Center for Bioethics and Department of Social Medicine, University of North Carolina at Chapel Hill, 333 S. Columbia Street, Campus Box 7240, Chapel Hill, NC 27599 USA; 2 UNC Project Malawi, Tidziwe Centre, Private Bag A-104, Lilongwe, Malawi; 3 Department of Health Behavior, UNC Gillings School of Global Public Health, University of North Carolina at Chapel Hill, 170 Rosenau Hall, 135 Dauer Drive, Chapel Hill, NC 27599 USA; 4 Institute for Global Health and Infectious Diseases, University of North Carolina at Chapel Hill, Bioinformatics Building, 130 Mason Farm Road, Chapel Hill, NC 27599 USA; 5 The Kennedy Institute of Ethics, Georgetown University, 3700 O Street Northwest, Washington, DC 20057 USA; 6 Berman Institute of Bioethics, Johns Hopkins University, 1809 Ashland Avenue, Baltimore, MD 21205 USA

**Keywords:** Pregnancy, Women’s views, HIV prevention, Clinical trials, Qualitative methods, Research ethics

## Abstract

**Background:**

The pressing need to expand the biomedical HIV prevention evidence base during pregnancy is now increasingly recognized. Women’s views regarding participation in such trials and initiating PrEP while pregnant are critical to inform evolving policy and best practices aimed at responsibly expanding evidence-based access for this population.

**Methods:**

We conducted 35 semi-structured interviews with reproductive-aged women in Malawi in the local language, Chichewa. Participants were HIV-negative and purposively sampled to capture a range of experience with research during pregnancy. Women’s perspectives on enrolling in three hypothetical HIV prevention trial vignettes while pregnant were explored, testing: (1) oral PrEP (Truvada) (2) a vaginal ring (dapivirine), and (3) a randomized trial comparing the two. The vignettes were read aloud to participants and a simple visual was provided. Interviews were audio-recorded, transcribed, translated, and coded using NVivo 11. Thematic analysis informed the analytic approach.

**Results:**

A majority of women accepted participation in all trials. Women’s views on research participation varied largely based on their assessment of whether participation or nonparticipation would best protect their own health and that of their offspring. Women interested in participating described power dynamics with their partner as fueling their HIV exposure concerns and highlighted health benefits of participation—principally, HIV protection and access to testing/treatment and ancillary care, and perceived potential risks of the vignettes as low. Women who were uninterested in participating highlighted potential maternal and fetal health risks of the trial, challenges of justifying prevention use to their partner, and raised some modality-specific concerns. Women also described ways their social networks, sense of altruism and adherence requirements would influence participation decisions.

**Conclusions:**

The majority of participants conveyed strong interest in participating in biomedical HIV prevention research during pregnancy, largely motivated by a desire to protect themselves and their offspring. Our results are consistent with other studies that found high acceptance of HIV prevention products during pregnancy, and support the current direction of HIV research policies and practices that are increasingly aimed at protecting the health of pregnant women and their offspring through responsible research, rather than defaulting to their exclusion.

## Background

Women are disproportionately burdened by the HIV epidemic in many regions, with infection rates for young women (15–24) almost double those of young men [1]. Further, women who are pregnant are at increased risk for HIV acquisition, due to both biological [2–4] and sociobehavioral factors [5–7]. Given high viremia levels and missed opportunities for testing and treatment, women who seroconvert during pregnancy have significantly increased rates of vertical HIV transmission relative to women who were living with HIV prior to becoming pregnant [8, 9].

For all of these reasons, pregnant women are among those most in need of access to safe and effective HIV preventives, or pre-exposure prophylaxis (PrEP). But evidence is limited regarding the safety, dosing, and efficacy of PrEP during pregnancy. Pregnancy has been an exclusion criteria for the vast majority of clinical trials of PrEP (e.g. HPTN 035, CAPRISA 004, MTN-003, Partners PrEP), contraception requirements for women of reproductive age are standard, and women who become pregnant are typically removed from the study product [10–14]. Because the data on these products during pregnancy are so limited, there are enduring questions about safety and efficacy, resulting at times in divergent policy and practice recommendations [15].

Truvada, a daily oral pill, is currently the only FDA-approved biomedical HIV preventive for women. Based on data from inadvertent pregnancies during oral PrEP trials, as well as pregnancy-specific HIV and hepatitis B treatment trials, evidence suggests that the medications in Truvada–tenofovir (TDF) and tenofovir–emtricitabine (TDF-FTC)—are safe for use during pregnancy, though further research has been called for [16]. Another promising PrEP modality, the dapivirine vaginal ring, is currently under review by the European Medicines Agency (EMA) under Article 58, a procedure which allows the EMA, in conjunction with the World Health Organization (WHO), to provide a scientific opinion on medicines for use in low-and middle-income countries for diseases of major public health interest. The EMA’s opinion is expected to inform and facilitate national level regulatory decision-making in many countries [17]. Although pregnant women have been excluded from trials to date, data from inadvertent pregnancies indicate no adverse effects of dapivirine vaginal ring use at periconception on pregnancy or infant outcomes [18], and studies prospectively including pregnant women are being considered.

Those working to address the evidence gaps around HIV prevention during pregnancy will need to understand what matters most to women who might consider enrolling in prevention trials when pregnant. There are some data regarding women’s views about participating in research on other interventions while pregnant [19]; a few recent studies have addressed women’s attitudes regarding the use of PrEP during pregnancy in clinical contexts [19, 20]; others have explored the experiences of women in serodiscordant couples who decided to continue on PrEP after becoming pregnant during an open label demonstration project [20–23]. However, no studies to our knowledge have assessed factors influencing women’s interest in participating in a HIV prevention clinical trial that involves initiating PrEP while pregnant. To address this gap, we elicited the views of Malawian women through a qualitative vignette study.

Malawi was an appropriate site to interrogate these questions for several reasons. Historically, Malawi has been a country on the forefront of research with pregnant women living with HIV in prevention of mother to child (PMTCT) trials, and was the first to implement Option B+, now the global standard of offering pregnant women lifelong daily single dose ART, regardless of CD4 count [24]. While these efforts have improved outcomes for HIV-infected women and their offspring, the HIV epidemic in Malawi disproportionately affects women, with young women bearing 70% of infections among young people aged 15–24 [25]. Together with a high fertility rate [26] and robust HIV research infrastructure, Malawi has been considered a potentially important site for prevention research *during pregnancy* and offers an important context to explore women’s views on participating in biomedical HIV prevention trials and initiating PrEP while pregnant.

## Methods

The data for this analysis were collected in partnership with UNC Project Malawi and as part of a larger project, Pregnancy and HIV/AIDS: Seeking Equitable Study (PHASES). We conducted qualitative, in-depth interviews using a semi-structured guide to assess the views of women who might be eligible to participate in HIV research during pregnancy about (1) their experiences with research; (2) selected rules governing the intersection of research and reproduction; and (3) their responses to theoretical vignettes describing research during pregnancy. The latter are reported here.

### Participants

Participants included in this analysis were all HIV-negative, and purposively sampled to capture a range of experience with research during pregnancy. UNC Project Malawi in Lilongwe is a study site for U.S. National Institute of Allergy and Infectious Diseases (NIAID) HIV/AIDS Clinical Trials Network studies, many of which involve women of reproductive age. Just over half of the sample (see Table 2) were either: (1) women who had previously participated in a biomedical HIV prevention trial during pregnancy, or (2) women who were denied enrollment in a biomedical HIV prevention trial due to pregnancy; women meeting either criterion are described here as “research experienced”. Research experienced participants were identified by Malawi based research outreach staff through existing trial participation records at UNC Project Malawi. For this purposively selected research experienced subsample, current pregnancy status was not an eligibility requirement. Outreach staff contacted the women by phone, provided a brief description of the study, and invited them to participate. Those who indicated interest were scheduled for an interview appointment in a private office used for research administration.

The remaining participants were a convenience sample of women who were currently pregnant, recruited from a local antenatal care clinic. Interviewers approached women at the clinic and provided a brief explanation of the study. If the woman expressed interest, she was given the options of either reviewing the informed consent information and completing the interview after her clinic appointment in a private, adjacent room, or scheduling a more convenient time to return. Recruitment was designed around clinic flow and based on the limited availability of the interviewers. As such, an overall response rate was not calculated.

As previously described [27], our objective in utilizing this sampling methodology was not to approximate a representative sample, but rather to surface and explore the range of issues and concrete considerations relevant to women who might be eligible to participate in studies while pregnant, that should inform discussions surrounding policy and best practices regarding the inclusion of pregnant women in clinical HIV prevention trials.

### Data collection

Data for this analysis are based on 35 in-depth interviews conducted with reproductive-aged women at risk for HIV in Lilongwe, Malawi. In-depth interviews were utilized instead of focus groups because the topic—considerations regarding participation in HIV-related clinical trials during pregnancy—is highly personal and potentially sensitive, and we wanted to deeply explore women’s considerations about participating in such research. Interviews were conducted in Chichewa by two trained, bilingual, local social behavioral scientists both experienced in qualitative HIV research (TW and CZ), using a semi-structured guide between August 2016 and April 2017 [27]. Female interviewers were purposefully selected to encourage the comfort and candor of participants.

Written informed consent was obtained prior to the interview, and women consented to being audio-recorded. Women also answered demographic questions and questions assessing their current pregnancy status, pregnancy history, and HIV testing/treatment history. Interviews lasted approximately 45–60 min. In line with recommendations by the National Health Science Research Committee of Malawi (NHSRC) at the time, participants were reimbursed 3500 Malawi Kwacha, the equivalent of $5 USD, for costs associated with participation (i.e., transportation). In the consent process, we described efforts to protect confidentiality, and assured participants that names and other identifying information would not be linked to their direct quotes in publications. The research was approved by the institutional review boards at the University of North Carolina (UNC) at Chapel Hill, Johns Hopkins University (JHU), and the National Health Science Research Committee of Malawi.

Interview guide development was informed by a review of the scholarly literature on women’s participation in research during pregnancy, interviews with HIV investigators exploring barriers to and facilitators of including pregnant women in clinical trials [28], and consultations with researchers and healthcare providers in the U.S. and Malawi. The interview guide was developed in English, and then translated into Chichewa by TW and CZ. It was then used in preliminary interviews, and revised to enhance cultural appropriateness, clarity, and flow.

As part of the interviews, we elicited women’s responses to the prospect of participating while pregnant in any of three hypothetical HIV prevention clinical trial vignettes, testing: (1) oral PrEP, (2) the vaginal ring, and (3) a randomized trial comparing the two (see Table 1). The use of succinct, standardized vignettes and subsequent probes allowed us to surface women’s initial receptiveness and considerations regarding participation within and across the trio of hypothetical HIV prevention research scenarios. The vignettes were read aloud to participants and consisted of a brief introductory overview of the purpose, design, participant requirements, and known safety data during pregnancy. Women were also provided a simple visual aid for each scenario.

**Table 1:** HIV biomedical prevention trial vignettes

1. Oral PrEP
You are pregnant. You get asked whether you would be willing to join a study to evaluate whether daily use of an anti-HIV pill can be safely used during pregnancy to prevent HIV. You will be asked to take the medication daily and to continue to take it for 6 months after you give birth. The medication has been tested in two different kinds of women who are a lot like you. It works in women who are not pregnant to reduce the chances of HIV infection. And it works for women who are pregnant but already have HIV, as a way to prevent them from passing HIV to their babies. But it has not been tested extensively in pregnant women like you who do not have HIV but want to protect themselves from infection.
2. Vaginal ring
You are pregnant. You get asked whether you would be willing to join a study to evaluate whether monthly use of a vaginal ring, which releases an anti-HIV medication, can safely prevent pregnant women from getting HIV. The ring is a flexible plastic device that you put in the vagina. You change it once a month. You will use it during pregnancy and for 6 months after giving birth. It seems to work in women who are not pregnant to prevent HIV. Also, the medicine in the ring has been used in pills for pregnant women who have HIV and is thought to be safe. But the ring itself has not been tested in pregnant women.
3. Randomized control trial (RCT): oral PrEP vs. vaginal ring
You are pregnant. You get asked whether you would be willing to join a study about the safety and effectiveness of the two prevention methods discussed above (the vaginal ring and the daily pill). No one knows if the ring or the daily pill will work better than the other. If you join the study, whether you get the ring or the pill will be decided by a process called randomization. When patients are randomized to one drug or the other, it makes it easier for the researchers to understand the study’s results. Randomization works something like a gumball machine with red and blue gumballs in it. If you get a red gumball, you get one medicine, and if you get a blue gumball, you get the other medicine. You don’t know whether you will get the red gumball or the blue gumball, but you know you will get a gumball and therefore some kind of medicine.

After each vignette was read, the interviewer would answer any questions the participant had about the scenario before proceeding. We then asked if women would participate in each vignette, and what was most important to them in making this decision. Additionally, participants were then told that, “Some people think about specific risks and benefits when they decide about participating in a study,” and asked if they thought about it this way. If participants responded affirmatively, we then probed specific risks and benefits to the baby and to the women themselves that they had considered.

### Analysis

Interviews were audio recorded. After each interview, the interviewer wrote a summary and recorded her impressions. Trained, local bilingual research staff experienced in transcription and translation both transcribed the Chichewa audio recordings following a standardized protocol, and then translated the transcripts into English. The interviewer reviewed the Chichewa transcript for accuracy prior to translation, and again after they were translated into English. Additionally, the US-based Project Director (KS) reviewed the interview summaries and English transcripts as they were produced and provided feedback to the interviewers. English transcripts were uploaded into NVivo 11 for analysis.

Codebook development was a collaborative process within the cross-cultural analytic team (KS, TW, EJ, and CZ) that began when the first 15 interviews were completed. Thematic analysis informed the analytic approach [29]. We familiarized ourselves with the data by reading and rereading the transcripts and summaries, and making notes of our impressions. Structural codes were first applied to organize the data by question and response. Content coding was then developed, with initial codes modified as we worked through the coding process with our research question in mind, and transcripts recoded as necessary. Throughout the coding process, the cross-cultural analysis team had extensive discussions about cultural and other meanings of responses. To enhance the cultural integrity and overall validity of the analysis, all transcripts were coded by at least one local researcher (TM, CZ). Additionally, to ensure intercoder reliability, 20% of the data were double coded, and any discrepancies were discussed until consensus was reached through re-coding or revising our understandings of the codes.

With NVivo 11 software, we extracted the text for the interview sections of interest, and utilized data display matrices to make comparisons within and across respondents and identify thematically and conceptually overarching themes. Sub-themes were grouped into themes based on thematic similarities, and the dataset was analyzed for the prevalence and breadth of identified themes. Summaries of each theme including exemplary quotes are presented. Data saturation was assessed and confirmed as coding progressed and no new themes were found in subsequent transcripts [30].

Demographics were self-reported by participants. Of note, the cultural concept of marriage in Malawi extends beyond the legal definition and includes co-habitating couples jointly raising children. Methods are also described in detail elsewhere [27].

## Results

Participant demographics are presented in Table 2. Overall, just over half of the sample was 25 to 34 years old, and 29% were less than 25 years. The majority of the sample either had a primary school education (46%), or had completed some secondary school (31%). A large majority described themselves as married (89%) and Christian (60% Protestant, 26% Catholic).

**Table 2:** Participant characteristics

Characteristic	Total n = 35	
	n	%
Age		
< 25	10	29
25–34	18	51
35–44	5	14
45+	2	6
Education		
None	5	14
Primary: Some to completed	16	46
Some secondary	11	31
Completed secondary	2	6
Post-secondary	1	3
Marital status		
Single	2	6
Married	31	89
Living with partner	–	–
Divorced or separated	1	3
Widowed	1	3
Number of pregnancies		
1	4	12
2–3	13	37
4+	18	51
Number of living children		
0–1	10	29
2–3	14	40
4+	11	31
Religion		
Christian		
Catholic	9	26
Protestant	21	60
Muslim	4	11
Other/not reported	1	3
Research experience during pregnancy		
Yes	19	54
No	16	46

### Overall initial receptiveness to participating in biomedical HIV prevention trial vignettes

The majority of women reported initial receptivity to joining the oral PrEP and vaginal ring trials described in the vignettes (Table 3). With few exceptions (n = 2), respondents agreed to participate in the randomized controlled trial (RCT) comparing oral PrEP and the vaginal ring if they had responded they would participate in trials specific to each. Five overarching themes were identified across vignettes: protecting the health of self and baby, power dynamics with partner, social influences, altruism and adherence requirements.

**Table 3:** Participants’ initial receptiveness to participating in biomedical HIV prevention trial vignettes

Participation	Total n = 35	
	n	%
Vignette 1. oral PrEP		
Would participate	28	80
Would not participate	6	17
Unsure/it depends	1	3
No response	0	0
Vignette 2. vaginal ring		
Would participate	24	69
Would not participate	10	29
Unsure/it depends	0	0
No response	1	3
Vignette 3. RCT: oral PrEP vs. vaginal ring		
Would participate	25	71
Would not participate	7	20
Unsure/it depends	–	–
No response	3	9

### Protecting the health of self and baby

Women’s views on research participation varied largely based on their assessment of which course of action—participation or nonparticipation—would best protect the health of themselves and their offspring. The most dominant theme by breadth and depth, women universally described this as their primary motivation. Women who were interested in participating in a vignette highlighted the health benefits conferred by participation—principally, HIV protection and access to testing/treatment and ancillary care, and perceived the potential risks of participation as low. Alternatively and less commonly, women who were uninterested in participating in a vignette highlighted the potential maternal and fetal health risks of the trial they wished to avoid, with some modality-specific concerns raised.

Many women perceived a risk of exposure to HIV during pregnancy, and saw the potential protection offered by these trials for both themselves and their future offspring as a significant benefit.

*“I can join because the pills can provide protection. Where you could have contracted the disease, you cannot contract it because of the pills.”*

Research inexperienced participant, oral PrEP vignette

*“What has made me accept (participation) is that I should give birth to a child who does not have the virus… we should protect the unborn child…”*

Research experienced participant, vaginal ring vignette

Another commonly mentioned benefit of participation was access to ancillary and/or higher quality care, which was also described as a way to protect oneself. A woman who previously participated in an HIV prevention study described problems experienced in standard care, and the superiority of care provided in the context of a clinical trial.

*“When you get pregnant you can have diseases in your body. You go to the hospital and they test you, they tell you that you have diseases… (A)t the hospital they tell you there is no medicine for this disease and they give you inappropriate medicine. So research like the one you want to start here, I have seen that it is very good … because we protect our bodies.”*

Research experienced participant, oral PrEP vignette

Participants particularly noted the benefits of being tested for HIV in the course of the clinical trial and knowing one’s status, with some describing the advantage of having quick access to ARVs if they seroconverted during a trial.

*“…(B)ut if you meet the doctor and get tested, he will tell you whether you have the virus or not and when you will start medicine… so the benefit is getting the blood tested… (T)he other benefit is taking medicine whilst the immunity is still high. If you are found with the virus, they tell you when to start taking medicine. It means I will be healthy for a long time.”*

Research experienced participant, oral PrEP vignette

Respondents also described the health education related to HIV prevention trial participants receive as enhancing their ability to protect themselves.

*“The benefit is there because it helps people to learn something, like if a person did not know that this way can help to prevent me from getting infected sexually…”*

Research experienced, RCT vignette

Additionally, many women saw participation in the trials as presenting low risks to their own health and that of their fetus. Notably, when asked how they weighed the risks and benefits of these vignettes, some women who agreed to participate expressed that they only thought of the benefits, not of the potential risks.

*“The benefit is that one of not getting diseases, protecting yourself. I did not think of the threats.”*

Research experienced, dapivirine vaginal ring vignette

Several women specifically cited the information provided in the oral PrEP vignette that the drug has been studied in pregnant women living with HIV and found to be safe as influencing their low risk assessment of participation.

*“I cannot talk of the risk because I have not taken the pills before, so maybe after I take them. But if the medicine was already tested on pregnant women and it had no risks, I cannot say I will find any.”*

Research inexperienced, oral PrEP vignette

In contrast and less commonly, participants who were not interested in participating in the trial vignettes described their decision as protecting themselves and their babies from the potential health risks of research. The primary reason women reported they would decline participation was linked to concerns around unknown side effects, risks to their personal health, or risks to their baby, including the risk of pregnancy loss, with some different concerns identified or emphasized across the modalities.

For the oral PrEP vignette, concerns were primarily around potential side effects and harm to the developing fetus or pregnancy loss.

*“The risk… is that maybe I don’t have the disease, if I take the medicine, I can start experiencing some things… falling sick unexpectedly, maybe dizziness, or seeing that your body is not looking the way it (usually) does, or maybe thinking that that if I am looking like this, what is the child inside me experiencing?”*

Research inexperienced participant, oral PrEP vignette

*“(M)aybe you take the medicine while you are pregnant and maybe the pregnancy can be lost.”*

Research experienced participant, oral PrEP vignette

With regard to the vaginal ring, several women expressed concerns about inserting something into their vaginas, and beliefs that the ring may be painful.

“*I am not interested in inserting things, that is why I feel this study is difficult.”*

*Research experienced, vaginal ring vignette*

*“The problem I might find, maybe urinating, can’t you feel pain? When having sex, can’t you feel pain?”*

Research inexperienced participant, vaginal ring vignette

Respondents also noted fears of potential fetal harm related to location of the ring, including sex causing adverse displacement of the ring, and possible entanglement with the baby during labor and delivery.

*“But the risk, can’t it be possible to find that the ring has moved, it has gone inside and has brought about another problem? … (Y)ou put it here and then it moves in, since you will be having sex with the husband and maybe he is making contact with it…. (I)f it moves and goes inside it means you will obviously need an operation. So you find that you have given birth to a child prematurely since they have operated on you because of the ring.”*

Research inexperienced participant, vaginal ring vignette

*“Let’s say you have started labor pains and you have come here at the hospital… It’s the same place the ring is inserted and the child comes out from. (T)he nurse will wonder what it is and by the time she realizes it will be too late.”*

Research inexperienced participant, vaginal ring vignette

### Power dynamics with partner

While health benefits were a major consideration in decision making, women’s assessment of such potential benefits as well as risks of research participation were importantly shaped by power dynamics with her partner. Some participants specifically articulated that their desire for HIV protection was motivated by concerns over their partner’s fidelity and their limited power in negotiating sexual activity, including unwelcomed, condomless intercourse.

*“I have enrolled in the research and I do not have the virus plus I am pregnant. It could be that my husband is being unfaithful, but if I am taking the medicine I will be protected from the diseases he might contract and I would also protect the unborn child.”*

Research experienced, oral PrEP vignette

*“I think this ring research is good because maybe some husbands are cruel and they would just want to have sex with you when you don’t expect it. So with the ring, you would be safe to say, ‘Even though he has done this, I am protected’.”*

Research inexperienced participant, vaginal ring vignette

Less frequently, and in the other direction, some described how the power dynamics with their partner would discourage their participation in the vignettes. Several research inexperienced women believed use of a HIV prevention modality through enrollment in a study would be difficult to justify to their partner, who may believe they are hiding their HIV positive status or accusing him of being HIV positive, potentially resulting in relationship problems they were unwilling to risk.

*“It can happen I start this method and things go wrong at home…. (my partner may think) maybe you have the virus, you are just hiding… maybe he cannot understand.”*

Research inexperienced participant, oral PrEP vignette

*“I would refuse (participation)… I would take the ring but maybe my husband would be wondering why I am wearing the ring… (H)e would be asking me why I am wearing the thing as though he has diseases.”*

Research inexperienced participant, vaginal ring vignette

### Social influences

Women also discussed ways in which their broader social networks would support or discourage participation in the research vignettes. Several research experienced women described the influence of friends who are participating in a trial as encouraging their participation. A woman who previously participated in a HIV prevention study explained how peers influenced her decision to enroll in the trial.

*(W)hen your friends are saying, ‘I joined a research study,’ you envy them. After you envy them you say, ‘I should not just be staying after my friends have left, I should go and also join.’…. So you follow your friends. You get here and you see that the things are quite important and they might help you in the future. So that is when you say I am also joining.”*

Research experienced participant, oral PrEP vignette

Others viewed their community and familial settings as deterring their participation. Several women described how inaccurate beliefs about research, or rumors about study procedures, can develop and spread in communities, which can perpetuate a sense of distrust in the research process among some.

*“It is a difficult study to join… Also, people tell others misconceptions about the study. ‘This is what happens’ so many people are afraid to follow such studies and what they think is that ‘If there are studies there, let the people conducting these studies also enroll, they should be doing them themselves.’”*

Research experienced participant, vaginal ring vignette

A participant even described her fear of being shunned by her family if she joined a research study.

*“(M)y relations, if I join the research… they can say maybe this one joined Satanism… It’s like that in the village.”*

Research inexperienced participant, oral PrEP vignette

When considering the oral PrEP vignette, some participants’ fear of being labeled HIV-positive extended beyond their partners to other family and community members, noting a lack of familiarity with the concept of using HIV antiretroviral drugs for primary prevention.

*“A person does not take drugs for a disease he is not suffering from. Every time you take them people will be wondering what drug you are taking. They will think they are HIV drugs/ARVs and won’t believe if you deny it.”*

Research inexperienced participant, oral PrEP vignette

### Altruism

Respondents shared that they were motivated to participate in order to help others and/or contribute to science. A woman described the risks to pregnant women who get sick and are without access to effective medicines as a motivation for her to participate.

“*What is really important to me is that I have seen a lot of women, for example a pregnant woman … at the village and she suddenly gets sick, let’s say malaria, but since she cannot access drugs, you find she is dead. But we need to do this research to find the right drugs for a pregnant woman… so that when they are sick they can take the drugs.”*

Research inexperienced participant, oral PrEP vignette

*“(W)hen you are pregnant… you are at risk. So if they test the medicine on me… in the future it helps other people.”*

Research inexperienced participant, oral PrEP vignette

### Adherence requirements

Several participants noted the appeal of the relative ease of the monthly placement of the vaginal ring as contrasted to the challenge of remembering to take daily oral PrEP.

*“(F)or some it will be difficult to take this medicine (oral PrEP) daily. But… since when you wear the ring your body does not feel differently, you just stay as you always are. Even a man does not know that there is a ring. But maybe the medicine will be harder because it is hard to take daily.”*

Research experienced participant, oral PrEP vignette

## Discussion

Our study surfaced the views of Malawian women at risk for HIV toward enrolling while pregnant in three hypothetical biomedical HIV prevention research studies using vignettes. The majority of women conveyed preliminary interest in participating while pregnant in the oral PrEP and vaginal ring studies and the RCT described by the vignettes. Five dominant themes with corresponding subthemes emerged from women’s descriptions of benefits and concerns about participating in the vignettes, some of which are specific to the modality: protecting the health of self and baby; power dynamics with partner; social influences; altruism, and adherence requirements.

The most prominent theme motivating women’s decisions surrounding vignette participation across participants was the desire to protect their health and that of their offspring, and the majority of participants believed that participating in the research vignettes was conducive to this aim. Women described a strong motivation to remain HIV-uninfected, both for their own health and that of their future offspring, mirroring previous findings [20–22]. Notably, no respondents said they were unconcerned about potential HIV infection during pregnancy. At the time of data collection, biomedical HIV preventives were not available in Malawi outside of a research context, so participation in the research vignettes would provide a benefit that is otherwise unavailable [31].

Concerns have been raised that access to biomedical HIV prevention products in a trial conducted in a context with high HIV prevalence and where PrEP is otherwise inaccessible may be so appealing to potential participants as to represent undue inducement [32]; this motivation may be even further heightened during pregnancy. However, classifying an inducement to participate as undue requires that it have a distorting impact on a participant’s ability to rationally weigh costs and benefits of participation [33]. Given that women’s perceptions of HIV infection risk during pregnancy are well supported, their strong desires for protection rational, and their expectations that the trial intervention will reduce risk plausible, the inducement to participate here is reasonable, not undue [34]. In particular, recent research has suggested the risk of HIV seroconversion may be elevated during pregnancy, likely due to both biologic and sociobehavioral factors [2–7]. Given that pregnancy may be a time of heightened risk of infection to the woman, and that seroconversion during pregnancy elevates the risk of vertical transmission [9], rather than questioning if women’s desire for protection may distort their decision making abilities, we may more appropriately consider the ethics of denying these women access to such trials [35].

HIV prevention was not the sole health benefit identified. Many participants, particularly those who were research experienced, described the desirability of the quality and breadth of care provided in a biomedical trial, which may be particularly salient during pregnancy. Prior research has found access to ancillary care to be a primary motivator in this setting for clinical trial participation [36], and ethicists have argued that such access in limited resource contexts is a legitimate consideration for participants and to some extent a duty for researchers to provide [37–40]. However, strong motivation to access ancillary care may also signal a potential blurring of the lines between research and clinical care in the eyes of prospective participants. It is thus important for participants to understand that the goals of research differ from the goals of care. For example, medical procedures may be performed not because they are standard of care or to benefit the participant, but as part of the study protocol. Our research, while not designed to address this topic, does raise important questions of how pregnancy might differentially affect motivation related to ancillary care access in research. Further research should explore and analyze ethical considerations related to decision making around trials which may be a point of access for antenatal care services otherwise unavailable.

Many women specified that their perceived risk of HIV infection was due wholly or partly to power dynamics with their partner, and described the trials as a way to protect themselves against HIV exposure during pregnancy due to partner infidelity. However, relationship power dynamics also discouraged participation for numerous research inexperienced respondents who expressed concerns that participation may result in tensions with their partner surrounding accusations of undisclosed HIV status—either the woman herself being accused or her partner feeling accused of being HIV-infected. In work previously published with this sample on the topic of paternal consent for research participation, some participants cited fear of relationship conflict, including fears of violence, as influencing their views [27]. Those with prior research experience during pregnancy did not mention this fear as a deterrent to participation, which may be either because their prior participation in HIV prevention research reduced this barrier, or it wasn’t an issue for them to begin with. Alternatively, given our recruiting methods [25], none of the research experienced women were currently pregnant, whereas all of the research inexperienced women were. Though we asked all women to imagine they were pregnant when responding to the vignettes, certain concerns, including potential relationship conflict over research participation during pregnancy, may have been more readily imaginable to those who were currently pregnant. These findings expand understanding of the wide range of ways in which relationship power dynamics can affect women’s orientations toward research participation during pregnancy in African settings [23], and suggest a potential influence of prior HIV prevention research experience during pregnancy on their perceptions of power dynamics with their partner surrounding study enrollment. Understanding this factor as both a potential motivator and potential deterrent may be helpful in future community engagement efforts around HIV and other sexually transmitted disease related research with pregnant women, particularly when engaging male partners.

Community engagement efforts for future research with pregnant women may also be informed by the modality specific concerns raised by participants in our study. While concerns raised are consistent with prior work with women considering HIV prevention options [20–22], fears of HIV stigma surrounding PrEP initiation may be heightened in the context of pregnancy and should be further explored. For example, some women expressed fears that participating in an oral PrEP trial will lead to assumptions by family and/or community members that she is HIV positive and engaged in risky sexual behaviors. Fear of this potential stigma may be heightened during pregnancy and carry greater social repercussions, when reliance on one’s social network can increase. Additionally, concerns about the physical effects of HIV prevention modalities may raise distinct concerns due to the presence of the fetus (e.g., reported concern for fetal entanglement with the vaginal ring).

Our findings are consistent with limited prior research examining research experienced and inexperienced healthy volunteers’ perspectives on clinical research protocols, in that the majority of considerations identified overlapped [41]. In addition to differences regarding the effect of power dynamics with their partner, other distinctions included the encouragement of friends as a supporting factor and the challenges of daily oral PrEP adherence as a dissuading factor only for research experienced women. These concrete and specific concerns appeared to result from past research experiences which provided context in considering hypothetical vignettes. While no women interviewed had prior experience in an oral PrEP trial, some had experience with other prevention modalities, which may have illuminated potential challenges of adherence. In contrast, research inexperienced women lacked such experience, making their decision making more theoretical.

Several respondents noted concerns that would affect adherence. Certainly, adherence to PrEP is a broad concern generally, and not only in pregnancy [42–44]. In response to the well documented barriers to PrEP adherence, significant research efforts have focused on how best to motivate and support uptake and adherence, and have informed development of the next-generation of preventives designed to lower such barriers. Accounting for modality-specific concerns, including those that might specifically affect adherence in pregnancy, will be important to the generation of clinically useful data and effective implementation of new preventives in pregnant women.

It is worth noting that many women were also positively oriented towards the RCT vignette. While concerns in the ethics literature around randomization during pregnancy have primarily centered around the ethics of randomizing pregnant women to placebo arms [45–47], others have suggested that women who are pregnant may be generally disinclined to randomization given aversion to uncertainty [48, 49] and the importance to women of control during pregnancy [50]. However, concerns around being randomized to one of two active trial arms were uncommon barriers to participation in this study. Only two of the women who responded positively to both the oral PrEP and ring trials were uninterested in the RCT. In contrast, three women who had refused either the oral PrEP or the ring trials responded positively to the RCT, noting that they would simply drop out of the study if they weren’t randomized to their preferred product. These findings suggest that assessing intent to remain in a RCT regardless of study arm assignment during the informed consent process may help to reduce attrition rates.

Despite prior concerns in the literature around pregnant women’s willingness to join biomedical research generally [51, 52], we found many women would be willing, and in some cases, highly motivated, to participate in HIV prevention trials in this context. These findings provide support for several vanguard HIV prevention studies with pregnant women currently underway or in development, including IMPAACT 2009, which examines oral PrEP use during pregnancy and postpartum, and MTN-042 (DELIVER), which plans to randomize pregnant women to oral PrEP or the vaginal ring during pregnancy in the Malawian and comparable settings [53, 54]. Work to surface women’s perspectives on PrEP use in the context of pregnancy is ongoing to support MTN-042 [55].

### Limitations

The purpose of our methodology was to surface women’s initial receptiveness, and perceived potential benefits of and concerns about participation in HIV prevention trials while pregnant. The brief vignettes provided an overview of the studies, and did not include the detailed information contained in a standard informed consent process including potential risks to participants; without this information, respondents did not commonly foresee potential risks. Thus the actual enrollment decision participants would make in real life scenarios cannot be predicted from our findings.

The methodology was designed to identify factors influencing women’s views on HIV prevention research participation during pregnancy in the Malawian context. The relatively small sample is not representative of all women at risk of HIV in Malawi, nor women of reproductive age more broadly, and the findings cannot be generalized to these populations. Just over half of our sample was research experienced during pregnancy. While the overall sample may not be representative of the range of women who might be candidates for research, our sampling approach allowed us to capture a range of prior experiences of research that might inform decisions about participation, from being denied enrollment in a study due to pregnancy, to being removed from a study product when pregnancy occurred many months into trial participation. We were also able to capture the views of some women who had not previously been involved in research. Together the groups are representative of a range of women who may be recruited for study participation in the future and therefore offer important perspectives to consider.

Finally, our study did not explore women’s views on participating in biomedical research while pregnant for the full range of HIV prevention products in the development pipeline, including long-acting injectable cabotegravir [56], and ultra-long-acting, removable dolutegravir [57]. These products offer potentially important advantages, including simplified adherence, and added discreetness. As these products are developed, future studies should continue to explore women’s opinions on using these modalities during pregnancy in both research and clinical contexts.

## Conclusions

In our sample of research-experienced and research inexperienced Malawian women, our findings offer a strong account of women’s desire and range of motivations for enrolling in HIV prevention research during pregnancy. Our results are consistent with those of other studies that found high acceptance of HIV prevention products during pregnancy [20–23]. Our findings support the current direction of evolving HIV policies and practices that are increasingly directed at protecting the health of pregnant women and their offspring through responsible research, rather than defaulting to their exclusion. Certainly, decisions about when and whether to include pregnant women in research raise a range of complexities. Yet given the high burden of HIV, the current unavailability of biomedical HIV prevention modalities to pregnant women outside of trials in many contexts, the social and medical advantages of a woman-controlled product to prevent HIV, and the need for pregnancy specific data, there is strong reason to continue to advance these important studies. Our study adds yet another important reason: the desire of women themselves to participate in research initiating PrEP and other preventives during pregnancy.

## Data Availability

The full dataset generated and analyzed during the current study are not publicly available to protect participant confidentiality. As this is a qualitative study with a population of women described geographically and demographically, making the full data set publicly available could potentially lead to the identification of participants. Our ethics approval was granted based on the strict confidentiality of the individuals consenting to participate. The minimal data set informing the findings is contained in the manuscript, included as participant quotations, attributed to pseudonyms, that support each theme. Data are however available from the authors on reasonable request and with permission of the institutional review board at the University of North Carolina (UNC) at Chapel Hill, and the National Health Science Research Committee of Malawi.
